# The Texas Eye, Ear and Throat Charity Hospital

**Published:** 1898-05

**Authors:** 


### The Texas Eye, Ear and Throat Charity Hospital.

We have received from the executive surgeon of this institution, Dr. H. L. Hilgartner, his second annual report to the board of trustees. This report shows that much good work has been done by this worthy charity. A total of 512 individual patients were cared for at the hospital, with an aggregate of 7,160 cases. During the year 319 surgical operations were performed by the staff surgeons. A strong plea is made for a larger building for the accommodation of patients, the present building being inadequate.

The executive surgeon gives due credit to the Board of Lady Managers for their energy and devotion, "the success of the internal economy of the hospital being entirely due to their faithful and self-sacrificing efforts."

**Dr. J. A. Davis,** of Austin, has been appointed resident physician in charge of the Oklahoma Insane Asylum, located at Norman, Oklahoma. Dr. Davis was for many years one of the physicians in charge of the Texas State Lunatic Asylum at Austin, hence has had much practical experience in the care and treatment of the insane. He is especially fond of this kind of work. The JOURNAL congratulates the doctor on his appointment, and the Oklahoma institution on securing his services.

